# NACC data: Who is represented over time and across centers, and implications for generalizability

**DOI:** 10.1002/alz.70657

**Published:** 2025-09-18

**Authors:** Kwun C. G. Chan, Fan Xia, Walter A. Kukull

**Affiliations:** ^1^ National Alzheimer's Coordinating Center Department of Epidemiology University of Washington Seattle Washington USA; ^2^ Department of Biostatistics University of Washington Seattle Washington USA; ^3^ Department of Epidemiology and Biostatistics University of California San Francisco San Francisco California USA

**Keywords:** Alzheimer's Disease Research Center, generalizability, National Alzheimer's Coordinating Center, recruitment, representativeness

## Abstract

**INTRODUCTION:**

Since 2005, the Alzheimer's Disease Research Centers (ADRCs) have recruited participants into the Uniform Data Set (UDS), but enrollment trends and center‐level differences remain underexplored. This study investigates temporal patterns and heterogeneity in recruitment across ADRCs, with implications for generalizability.

**METHODS:**

Using data from the National Alzheimer's Coordinating Center (NACC), we assessed trends and between‐center variation in baseline characteristics, including age, sex, race, education, clinical diagnosis, referral source, family history, and co‐participant relationship.

**RESULTS:**

All characteristics except sex and family history showed directional shifts over time. Substantial between‐center heterogeneity was observed in all variables examined.

**DISCUSSION:**

Temporal changes and site‐level variability in participant profiles highlight challenges and opportunities for generalizing findings from UDS data. Although not nationally representative, statements about generalization could often be made using UDS data, with strengthened inferences and enhanced transparency through analytic approaches such as sensitivity analysis or meta‐analytic techniques treating centers as separate studies.

**Highlights:**

- The National Alzheimer's Coordinating Center (NACC) Uniform Data Set has enrolled participants for 20 years across more than 40 centers.
- We identified temporal trends and site‐level variation in participant characteristics in the initial visit.
- Despite being a volunteer sample, modern epidemiologic and biostatistical approaches can help assess and enhance the generalizability of scientific findings derived from NACC data.

## 1 BACKGROUND

Since 2005, the National Alzheimer's Coordinating Center (NACC) has collected longitudinal clinical measurements and diagnostic data from participants enrolled in the Uniform Data Set (UDS). While demographic summaries have been reported for early recruitment^1^ and the launch of Version 3 of the UDS,^2^ comprehensive evaluations of temporal patterns and between‐center heterogeneity remain limited.

Despite widespread use of NACC UDS data in research, concerns about the external validity of findings derived from these data persist,^3^, ^4^ and from observational cohorts more generally.^5^ A common view is that the generalizability of research findings hinges on the representativeness of study populations and the unbiasedness of recruitment strategies.^6^ Since each Alzheimer's Disease Research Center (ADRC) recruits participants through a mix of sources, such as clinician referral and community‐based recruitment, the NACC UDS data is best regarded as a volunteer sample without a specific sampling frame and is not a representative sample of the broader U.S. older population. A recent study quantified the discrepancies in many participant characteristics between NACC UDS and the Health and Retirement Study (HRS), which is nationally representative.^7^

Recruitment methods vary across ADRCs, reflecting diverse research priorities. A recent study used the proportion of cognitively normal participants at the initial UDS visit as a proxy for recruitment bias in ADRC autopsy studies.^8^ They showed that this proxy measure explains the difference seen in non‐AD pathologies and highlighted the importance of considering recruitment‐based heterogeneity in analyses.

**RESEARCH IN CONTEXT**

1. **Systematic review**: The authors reviewed the literature using search engines such as PubMed. Previous studies provided snapshots of recruitment characteristics of National Alzheimer's Coordinating Center (NACC) Uniform Data Set (UDS) data, and there is a lack of description of the recruitment heterogeneity across Alzheimer's Disease Research Centers (ADRCs). We also reviewed relevant literature in epidemiology on selection bias, representativeness, and generalizability.
2. **Interpretation**: Our results indicate that the NACC is a dynamic and evolving cohort, with substantial variability in recruitment characteristics across centers. We highlight both the challenges and opportunities for generating generalizable knowledge from NACC data.
3. **Future directions**: Greater efforts are needed to document center‐specific recruitment practices and their evolution over time. Future research should focus on understanding the transportability of estimates, identifying mechanisms of selection bias for various exposures and outcomes, and developing or applying advanced analytical methods, such as sensitivity analyses and meta‐analytic techniques, to enhance generalizability.

Further complicating generalizability is the shifting demographic landscape of the aging U.S. population. By 2030, one in five Americans will be aged 65 or older.^9^ The racial and ethnic composition of the older population is becoming more diverse, with substantial projected increases in racial backgrounds that historically have had a higher dementia prevalence but have been underrepresented in research studies.^10^ Educational attainment, a key protective factor against cognitive decline, has changed markedly across birth cohorts.^11^

Understanding how recruitment characteristics have evolved over time and across centers is crucial for assessing the applicability of research findings from NACC UDS data to broader or specific populations. This paper seeks to address the gap in knowledge by characterizing changes in recruitment over two decades and quantifying between‐center heterogeneity in baseline characteristics. We also outline methodological strategies and future directions to enhance generalizability.

## 2 METHODS

Data from NACC UDS were used in this study. The analytic sample was extracted from the September 2024 data freeze, which contains participants recruited between 2005 and 2024. Since the UDS contains data from past and present ADRCs, and new ADRCs are being funded over time, we restrict our analysis to centers with at least 3 years of participant recruitment to improve the stability of findings. We focus on participant characteristics obtained during the initial UDS visit.

The characteristics being studied were age, sex, race, education, clinical diagnosis, referral source, family history, and co‐participant relationship. Age at baseline visit was categorized into less than 60, 60–69, 70–79, and 80 or more. Self‐reported race was used, with three categories: White, African American, and others (including Indigenous Americans, native Hawaiian, Asian, and multi‐racial participants). We combined multiple race categories as others because each has a very small cell count across enrollment years or centers. Education is categorized into 12 years or less, 13‐16 years, 17‐18 years, and 19 years or more, which generally corresponds to high school, bachelor's degree, master's degree, and doctorate degree. The clinical diagnosis has four categories: normal cognition, impaired‐not‐MCI (mild cognitive impairment), MCI, and Dementia. The definition of referral type changed in version 3 of UDS, and we considered a binary variable for provider referral based on the NACC derived variable NACCREFR, which includes referral by ADRC or other research study clinicians, staff, investigator, as well as nurse, doctor, or other health care provider in UDS version 1.2 and 2.0, and clinician and clinic sample in UDS version 3.0. The NACC UDS data do not include a more detailed version of the referral source. Although the way referral source is recorded changed with the introduction of UDS version 3.0, no systematic or center‐wide changes to recruitment practices were proposed as part of its implementation. We assessed family history using the indicator of at least one first‐degree family member with cognitive impairment. Co‐participants’ relationship to the participants was categorized into spouse, child, and others.

We used the multinomial Cochran‐Armitage test for trend^12^ to evaluate the change in recruitment characteristics over time, and the Chi‐square test of homogeneity for assessing inter‐center variation in baseline characteristics. A nominal significance level of 5% was used. Statistical analyses were performed using R version 4.4.3.[Fig alz70657-fig-0001]

## 3 RESULTS

A total of 42 past and present ADRCs were included in the analysis, with a total of 51,984 participants recruited between 2005 and 2024. Among the ADRCs, 10 had 9 years or less of recruitment, 9 had recruitment of 10–19 years, and 23 had recruitment for 20 years (since the beginning of the UDS data). The number of participants recruited from each site ranged from 96 to 3249, with a median of 1188.

**FIGURE 1 alz70657-fig-0001:**
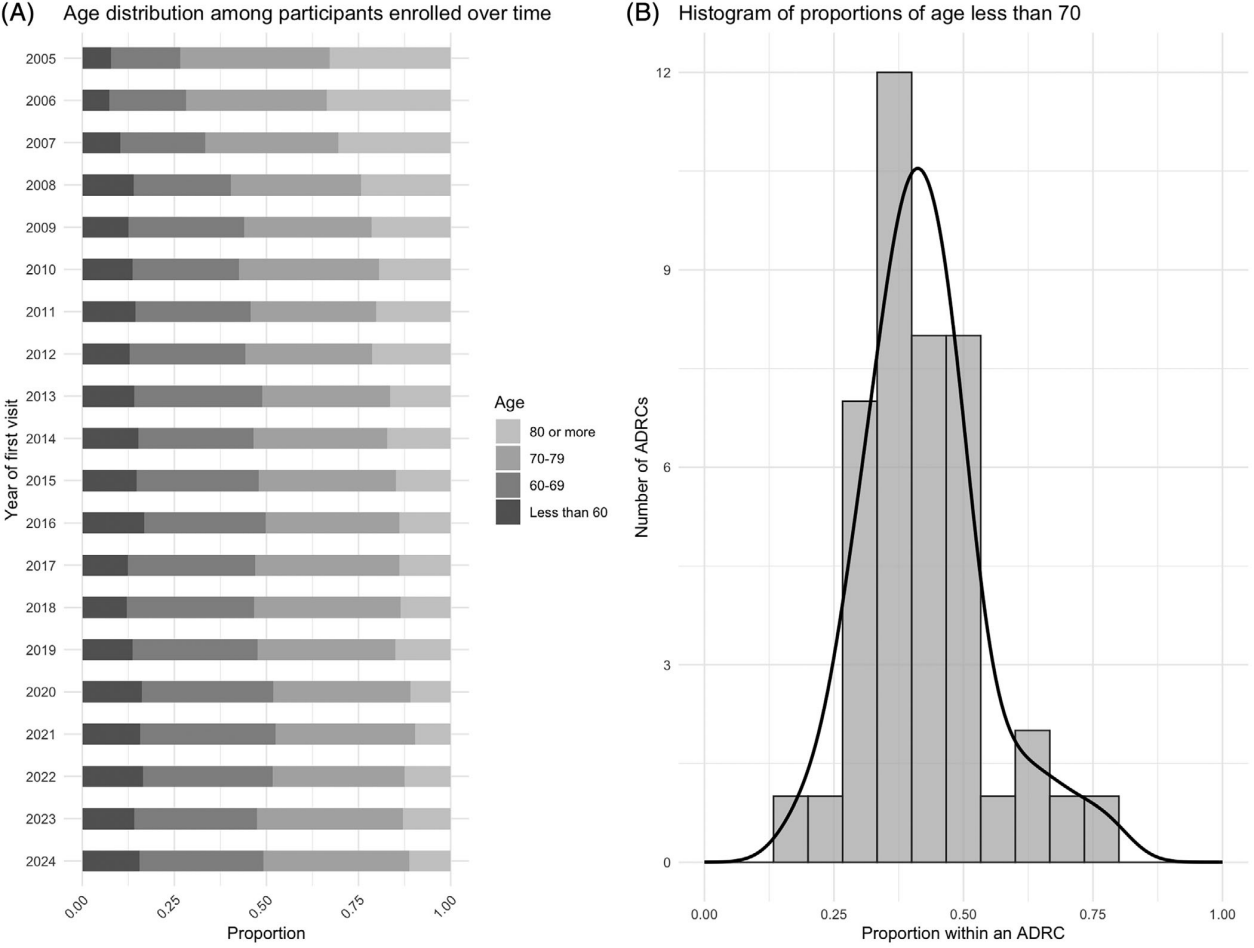
Age at enrollment over time and across centers.

**FIGURE 2:**
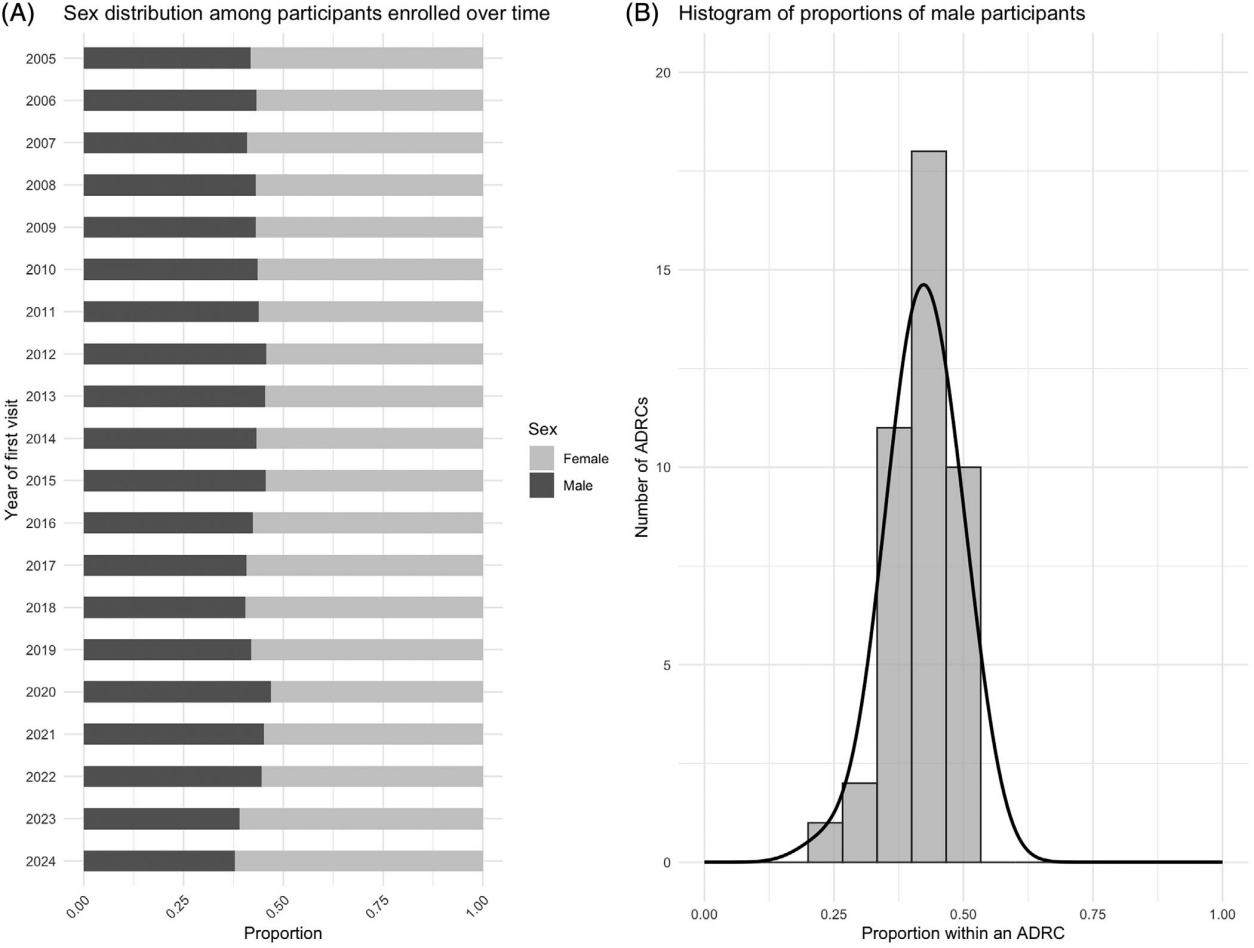
Sex distribution over enrollment time and across centers.

**FIGURE 3:**
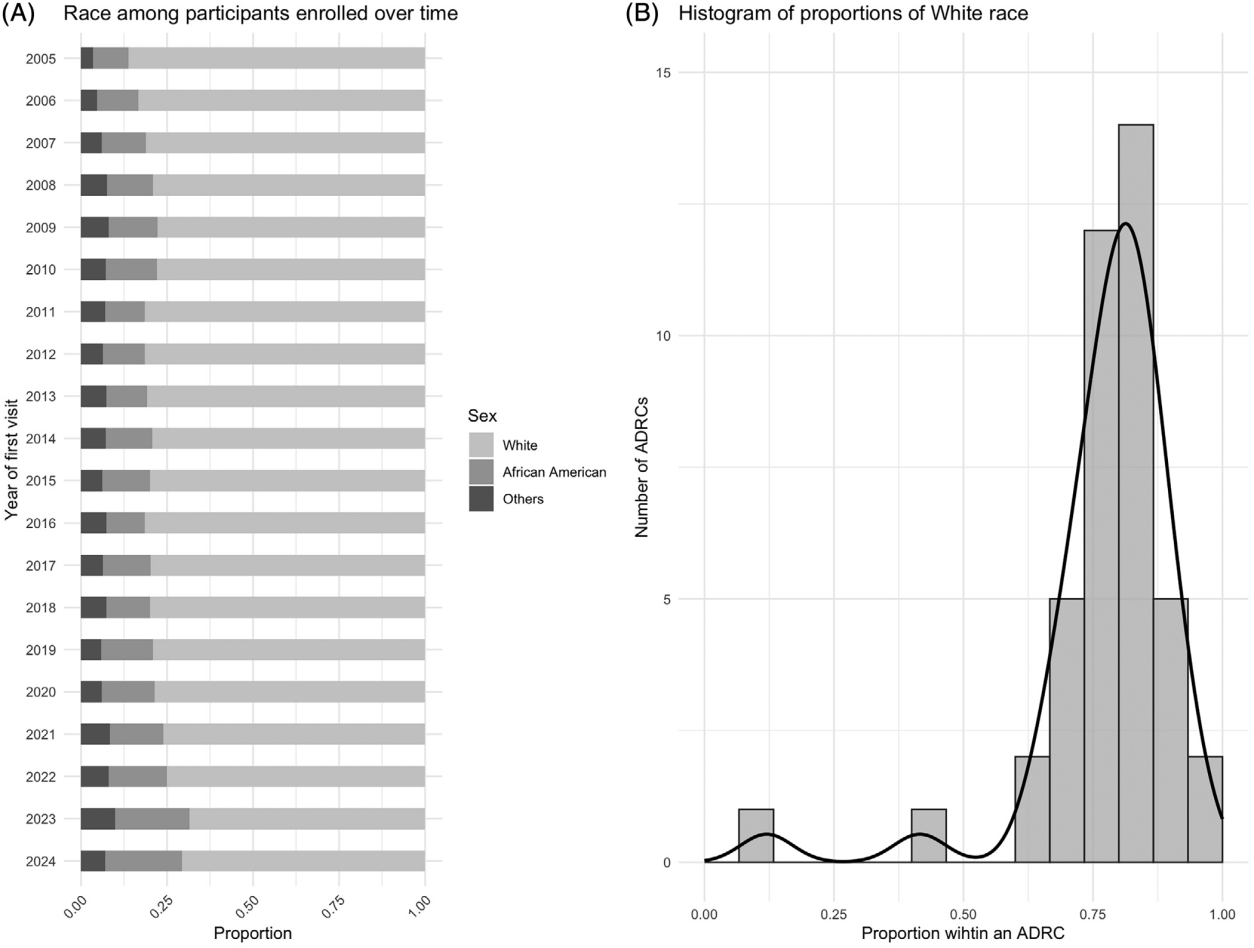
Race distribution over enrollment time and across centers.

**FIGURE 4:**
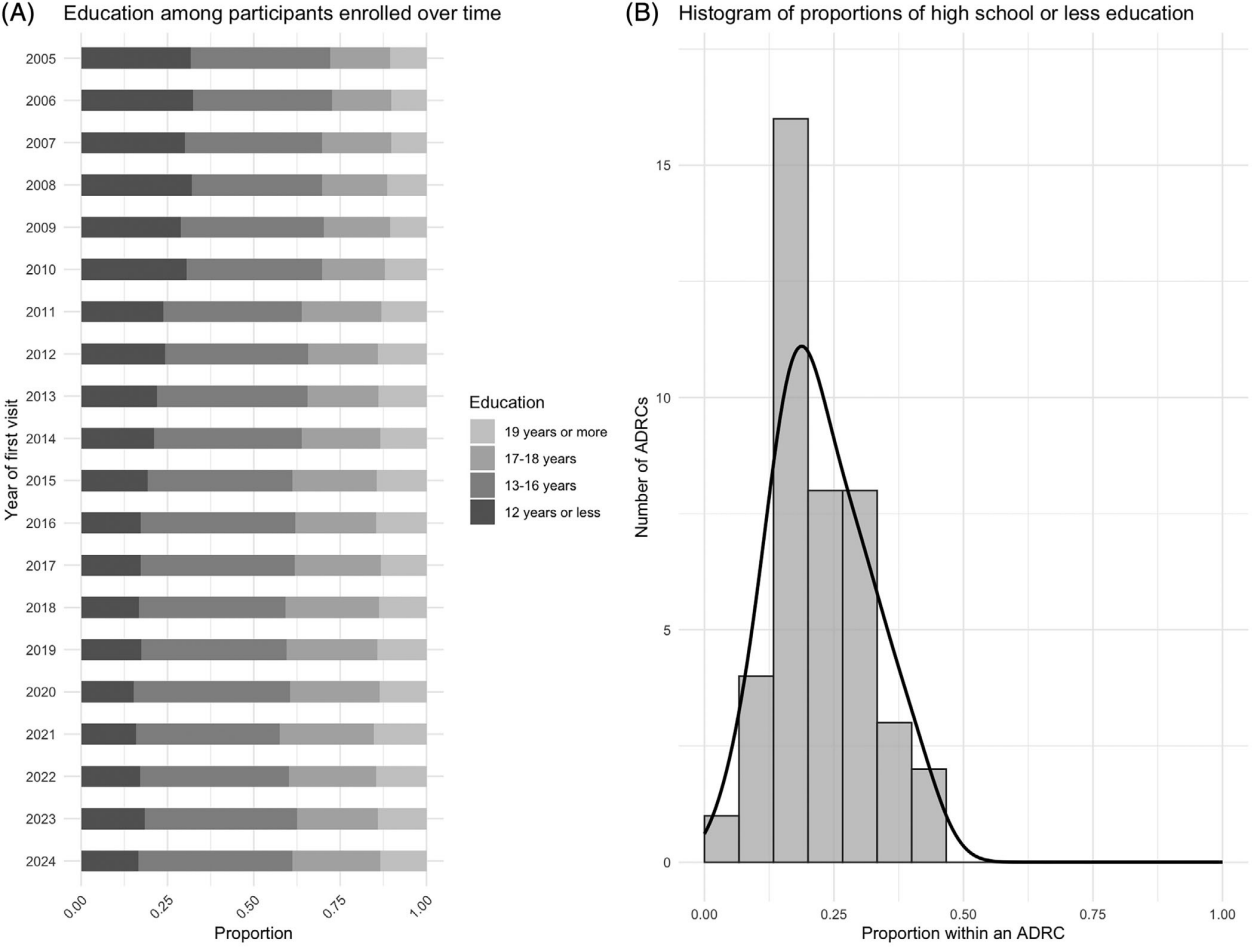
Education distribution over enrollment time and across centers.

**FIGURE 5:**
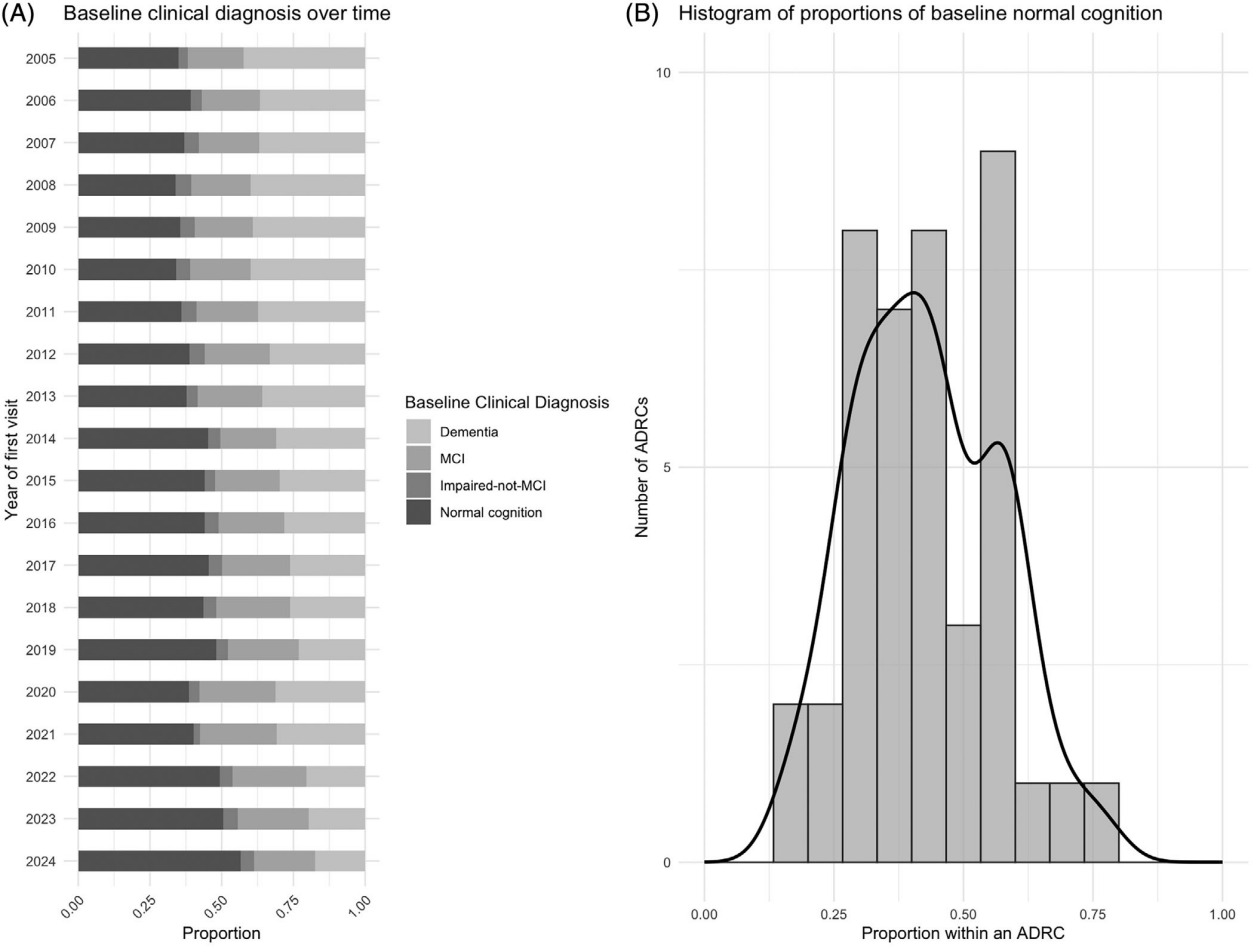
Baseline clinical diagnosis over time and across centers.

**FIGURE 6:**
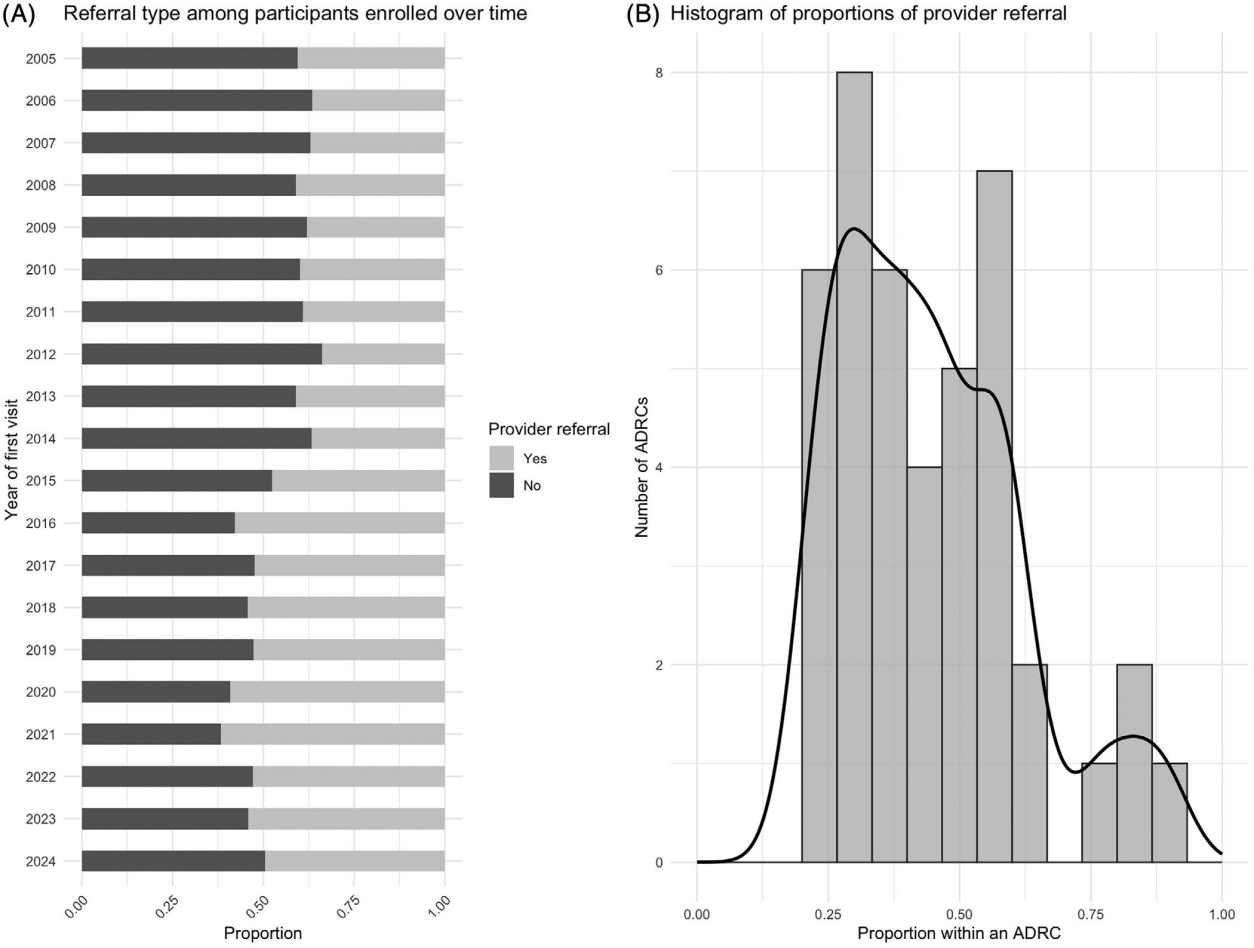
Provider referral over time and across centers.

**FIGURE 7:**
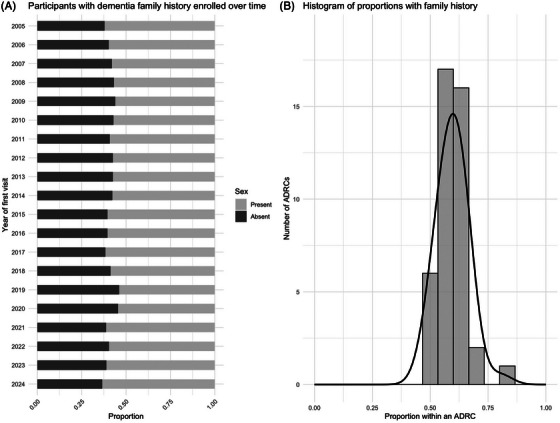
Family history over time and across centers.

**FIGURE 8:**
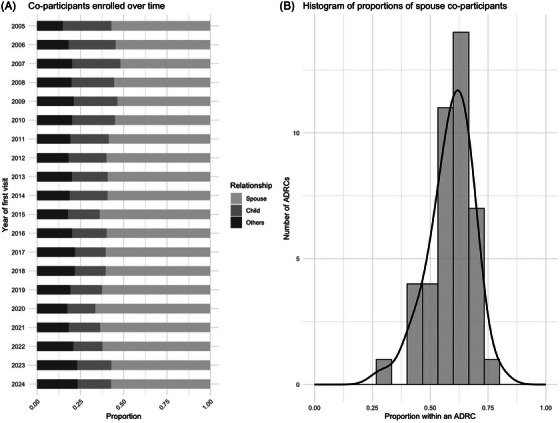
Co‐participant relationship over time and across centers.

Figures 1, 2, 3, 4, 5, 6, 7, 8 display the distribution over time and across centers for age, sex, race, education, clinical diagnosis, referral source, family history, and co‐participant relationship. Early in the history of the UDS, recruitment predominantly focused on older adults. More recent enrollment has included a broader age distribution, with increasing numbers of participants younger than 70 years at baseline. The trend test rejects a null hypothesis of no directional shift in age distribution over time (*p* < 0.001). The proportion of participants under age 70 at the baseline visit ranges from 19% to 78% across centers, with the median being 41%. There is a statistically significant difference in age distribution across centers (*p* < 0.001).

Overall, male participants comprised 43% of the total participants, and there was no significant time trend detected (*p* = 0.14). The proportion of male participants, however, differed significantly across centers (*p* < 0.001), ranging from 23% to 53%.

There was a modest but measurable change in the racial composition. While most ADRCs recruited predominantly White participants and continued to do so, the proportion of African American and other racial/ethnic minorities had increased over time (*p* < 0.001). Except for two centers, the proportion of White participants was between 65% and 95%. The proportion of White participants in the other two centers was substantially lower (12% and 42%, respectively). The proportion of White participants differed significantly across centers (*p* < 0.001).

The proportion of participants with a high school education or less declined over time. The trend test rejected a null hypothesis of no directional shift in educational attainment over time (*p* < 0.001). Some centers draw predominantly from highly educated populations, while others have broader educational representation. The proportion of high school or less education ranged from 6% to 42%, and differed significantly across centers (*p* < 0.001).

Over time, the new participants were more likely to be cognitively normal at enrollment (*p* < 0.001), but the distribution of baseline clinical diagnosis differed substantially across centers (*p* < 0.001). The proportion of normal cognition at baseline ranged from 18% to 76%. Some centers recruit over half of their participants with a baseline MCI diagnosis.

Healthcare provider referrals constituted a larger share of new enrollments over time (*p* < 0.001), although the increase appeared to peak in 2021. There is substantial heterogeneity according to referral type across centers (*p* < 0.001), with centers having as little as 20% up to 90% provider referral.

Overall, 58% of participants had at least one first‐degree relative with cognitive impairment, with no significant time trend detected (*p* = 0.21). The proportion varied substantially across centers (*p* < 0.001), ranging from 48% to 80%.

Trend analyses indicated a shift in the co‐participant's relationship to newly enrolled participants over time (*p* < 0.001). The proportion of child co‐participants declined, while the proportions of spouse and other co‐participants fluctuated over time. There was also substantial heterogeneity in co‐participant relationships across centers, with the proportion of spouses serving as co‐participants ranging from 29% to 79%.

## 4 DISCUSSION

The observed changes in recruitment characteristics over time possibly reflect evolving research priorities at ADRCs. The early recruitment emphasis aligned with early goals of characterizing clinical phenotypes of AD among the symptomatic older adults. The shift in baseline age and distribution of initial clinical diagnosis may reflect expanded interest in early detection, prevention, and preclinical stages of AD. The increase in the proportion of non‐White participants reflects the broader public outreach and engagement.

The NACC cohort is a large multi‐center effort, with data collection requiring a balance between multiple objectives, and allowing a wide variety of scientific questions to be addressed. The absence of uniform recruitment criteria across ADRCs creates a patchwork of population samples, which complicates efforts to derive generalizable conclusions. To support a wide range of scientific questions, recruitment strategies across ADRCs intentionally target heterogeneous subpopulations with diverse demographic and clinical characteristics. As a result, the resulting samples are not representative of the local regions or of the U.S. population, which limits generalizability in the traditional statistical sense. However, the diversity embedded in these samples broadens the relevance and scope of generalizable insights. Future effort is needed to better document center‐specific recruitment process over time to understand the evolution of the NACC cohort.

Representativeness is a sufficient but not necessary condition for generalizability, although it has historically been treated as both.^13^ Representativeness allows for an accurate snapshot of population characteristics, which may or may not be transportable to a different population or to a different time.^14^ Recent literature emphasizes transporting findings from non‐representative samples to target populations using weighting or covariate adjustment, but these methods typically rely on strong assumptions, such as overlap and the availability of common covariates, to capture all the selection biases.^15^ These assumptions may not hold between NACC UDS data and representative surveys such as the Behavioral Risk Factor Surveillance System (BRFSS) or HRS. Moreover, current transportability approaches typically do not account for changes in the source and target populations over time.

Generalizability, on the other hand, often targets the exposure–outcome association, that is, causal (e.g., due to a hypothesized biological mechanism) and invariant between populations and across time, and typically does not require representativeness for consistent estimation.^13^ Even when heterogeneous exposure effects are expected, representativeness to the general population is still not required and could be counterproductive. For instance, a sub‐population in which the effect is more pronounced may have a limited representation in the sample to achieve representativeness, while leading to a potential loss of statistical efficiency. Typically, sufficient variation in exposures matters more than population‐level representativeness, and studies therefore often intentionally target underrepresented exposure groups of interest.

Observational studies such as the NACC data are affected by selection bias, which can be broadly defined as any instance in which the parameter of interest in a target population differs from the parameter estimated from data available for analysis.^16^ The magnitude and direction of bias depend on both the specific scientific question and the actual recruitment mechanism. For example, suppose we aim to estimate the association between depression and AD in the general older adult population. Selection bias may arise if individuals within both AD and depression are more or less likely to be recruited when compared to AD cases without depression. In memory clinics with a strong focus on neuropsychiatry, AD cases with depression may be selectively referred and enrolled, resulting in over‐representation and inflating the association. In contrast, in community samples, individuals with both AD and depression may be less likely to be recruited and the association could be attenuated. In general, there is a variety of situations in which this can happen due to sample selection, such as when enrollment depends on factors that are upstream of the exposure in the causal pathway, lie between the exposure and outcome, or are downstream of both.^15^, ^17^ However, there is limited awareness of the various form of selection bias and few analytical methods available for correction, except in cases where selection is due to observed confounders and there is no effect heterogeneity due to unmeasured factors. In this case, weighting or regression adjustment based on observed covariates can theoretically remove the bias, but the strong assumptions required for such adjustments is often viewed as impossible in practice. On the other hand, the conclusion of a well‐powered study may be overturned by weak, unmeasured confounders. Since NACC has a large sample size, it often has high power to reject certain null hypotheses even when the effect size is small. Sensitivity analysis can be performed to quantify the minimum strength of the unmeasured confounder needed to remove an observed small but significant effect^18^, ^19^ and is a useful tool to quantify the strength of evidence in observational studies.

Center‐level effect heterogeneity can be examined by hierarchical models or a meta‐analysis framework, treating each center as a study. This approach is feasible because the number of ADRCs is large, and many of them have large sample sizes and long follow‐up periods. Ignoring center‐level effect heterogeneity often leads to under‐estimated standard error and can erroneously reject the null hypothesis due to inflated Type I error. In addition, inclusion or exclusion criteria differ across centers, and sometimes even within centers due to enrollment into affiliated studies. However, there is a lack of transparent documentation of these differences. This issue is analogous to what is commonly encountered in clinical trials, where meta‐analytic methods are routinely applied.^20^ Accounting for effect heterogeneity in estimation may help identify specific centers or omitted effect modifiers that warrant further investigation. Moreover, because recruitment strategies are decentralized, the mechanisms and consequences of selection bias likely differ across centers. In such cases, one can employ meta‐analytic techniques for sensitivity analysis of center‐specific estimates, which could provide more robust conclusions than those drawn from a single observational study design, which may be subject be a particular, but often unknown, form of selection bias.^21^, ^22^

## CONFLICT OF INTEREST STATEMENT

The authors report no conflicts of interest. The funders had no role in study conception, design, or writing of this manuscript. Author disclosures are available in the supporting information.

## CONSENT STATEMENT

Research using the NACC data was approved by the University of Washington Institutional Review Board. All contributing ADRCs are required to obtain informed consent from their participants and maintain their own local IRB reviews and approvals prior to submitting data to NACC.

## Supporting information



Supporting Information
